# HIV protease cleaves the antiviral m^6^A reader protein YTHDF3 in the viral particle

**DOI:** 10.1371/journal.ppat.1008305

**Published:** 2020-02-13

**Authors:** Denise Jurczyszak, Wen Zhang, Sandra N. Terry, Thomas Kehrer, Maria C. Bermúdez González, Emma McGregor, Lubbertus C. F. Mulder, Matthew J. Eckwahl, Tao Pan, Viviana Simon

**Affiliations:** 1 The Graduate School of Biomedical Sciences, Icahn School of Medicine at Mount Sinai, New York, New York, United State of America; 2 Department of Microbiology, Icahn School of Medicine at Mount Sinai, New York, New York, United States of America; 3 Department of Chemistry, The University of Chicago, Chicago, Illinois, United State of America; 4 Global Health Emerging Pathogens Institute, Icahn School of Medicine at Mount Sinai, New York, New York, United State of America; 5 Department of Biochemistry and Molecular Biology, and Institute for Biophysical Dynamics, The University of Chicago, Chicago, Illinois, United State of America; 6 Division of Infectious Diseases, Department of Medicine, Icahn School of Medicine at Mount Sinai, New York, New York, United State of America; Fred Hutchinson Cancer Research Center, UNITED STATES

## Abstract

N^6^-methyladenosine (m^6^A) is the most abundant HIV RNA modification but the interplay between the m^6^A reader protein YTHDF3 and HIV replication is not well understood. We found that knockout of YTHDF3 in human CD4+ T-cells increases infection supporting the role of YTHDF3 as a restriction factor. Overexpression of the YTHDF3 protein in the producer cells reduces the infectivity of the newly produced viruses. YTHDF3 proteins are incorporated into HIV particles in a nucleocapsid-dependent manner permitting the m^6^A reader protein to limit infection in the new target cell at the step of reverse transcription. Importantly, HIV protease cleaves the virion-incorporated full-length YTHDF3 protein, a process which is blocked by HIV protease inhibitors used to treat HIV infected patients. Mass-spectrometry confirmed the proteolytic processing of YTHDF3 in the virion. Thus, HIV protease cleaves the virion-encapsidated host m^6^A effector protein in addition to the viral polyproteins to ensure optimal infectivity of the mature virion.

## Author summary

The human transcriptome contains a large number of post-transcriptional modifications such as N6-methyladenosine (m^6^A). Several recent studies indicate that the HIV RNA contains numerous m^6^A modifications but their impact on viral replication (e.g., antiviral or proviral) remains controversial. Here we report that the m^6^A reader protein YTHDF3 is incorporated into HIV particles in a nucleocapsid-dependent manner and reduces viral infectivity in the next cycle of infection. Importantly, we show that HIV protease cleaves the virion-incorporated full-length YTHDF3 protein, a process which can be blocked by FDA-approved HIV protease inhibitors. Mass-spectrometry analyses confirmed the proteolytic processing of YTHDF3 in the virion and identified at least two distinct cleavage sites. These results point to virus incorporated YTHDF3 acting as a regulator of HIV biology if left unchecked by the HIV protease.

## Introduction

Post-transcriptional modifications of the transcriptome, such as N^6^-methyladenosine (m^6^A), are globally referred to as epitranscriptome [1–4]. m^6^A is a dynamic and reversible RNA modification involved in mRNA splicing, stability, localization, and translation [5–8]. m^6^A modifications have also been identified within the RNA genomes as well as transcripts of several viruses, including Influenza A virus, adenovirus, Rous sarcoma virus, hepatitis C virus, Zika virus, Dengue virus, West Nile virus, Yellow fever virus, and HIV-1 (HIV) [9–16].

While writer and eraser proteins add or remove m^6^A from the mRNA transcripts, reader proteins fulfill effector functions. m^6^A modifications that result in temporally-controlled burst of protein synthesis and mRNA decay, require the interaction with the YTHDF1-3 reader proteins [17]. YTHDF1 promotes translation efficiency of m^6^A-modified mRNA by recruiting and interacting with translation initiation factors and facilitating ribosome loading [18]. YTHDF2 promotes decay of m^6^A-modified mRNA by directing transcripts to cytoplasmic processing bodies [5]. Interestingly, YTHDF3 is capable of promoting both translation and decay of m^6^A-modified target transcripts [17]. Compared to YTHDF1/2, YTHDF3 has the strongest affinity for m^6^A-modified RNA [19]. YTHDF3 also binds to YTHDF1 and YTHDF2 in an RNA-dependent manner, which modulates binding specificity to target transcripts, as well as enhances function of YTHDF1 or YTHDF2 [17, 20]. YTHDF3 has been proposed to be the first reader protein to interact with m^6^A-modified transcripts in the cytoplasm acting as a gatekeeper by fine-tuning YTHDF1 and YTHDF2 access of target transcripts [17].

Our understanding of the role of m^6^A and its reader proteins in the context of HIV-1 life cycle remains incomplete. During HIV infection, the overall amount of cellular m^6^A modifications is increased in an envelope-mediated and HIV replication-independent process [11, 21]. HIV genomic RNA (gRNA) contains multiple m^6^A modifications [9, 11, 12], which positively affect virus replication by enhancing nuclear export of m^6^A-modified viral RNA and increasing viral gene and protein expression [9, 11]. Yet the exact roles for the m^6^A readers during HIV infection remain unclear. Indeed, the three m^6^A reader proteins, YTHDF1-3, play both a negative and positive role in HIV replication. While it is well established that YTHDF1-3 bind to HIV RNA transcript in infected cells [9, 12, 19], there are conflicting reports on whether YTHDF1-3 expression enhances or limits HIV infection. YTHDF1-3 overexpression in virus-producing cells was shown to enhance overall virus production [19] with YTHDF2 depletion also reducing HIV Gag production [12]. YTHDF1-3, however, also negatively regulate HIV infection at an early step of the viral life cycle such as viral reverse transcription [9] by a yet unknown mechanism.

We reasoned that some of the conflicting results in the context of HIV replication could be due to the fact that m^6^A modifications influence not only the translation of HIV genes (RNA→protein) but also HIV cDNA synthesis (RNA→DNA). We hypothesized that m^6^A reader proteins could both positively and negatively affect different steps in the viral life cycle. Here we investigated how the m^6^A reader protein YTHDF3 impacts the early steps of the HIV life cycle [9, 11, 12, 19] in the next round of infection. We found that the YTHDF3 protein is incorporated into egressing virions in a nucleocapsid dependent manner, limiting the efficiency of the next round of infection. Overexpression, complementation, and knock-out experiments in T cells underscore that YTHDF3 acts as a negative regulator of HIV particle infectivity. Importantly, our experiments show that HIV evolved a mechanism to limit the antiviral activity of YTHDF3. HIV protease cleaves virion-incorporated YTHDF3 at distinct sites; a process which is blocked by HIV protease inhibitors. Our studies suggest that endogenous YTHDF3 acts as a negative regulator of HIV if left unchecked by the HIV protease during virion maturation. Thus, our results uncovered a previously unrecognized mode of action of the currently approved FDA approved protease inhibitors and point to a novel antiviral strategy by which negative regulators of viral replication are antagonized.

## Results

### YTHDF3-deficient T cells are more susceptible to HIV infection

**Fig 1:**
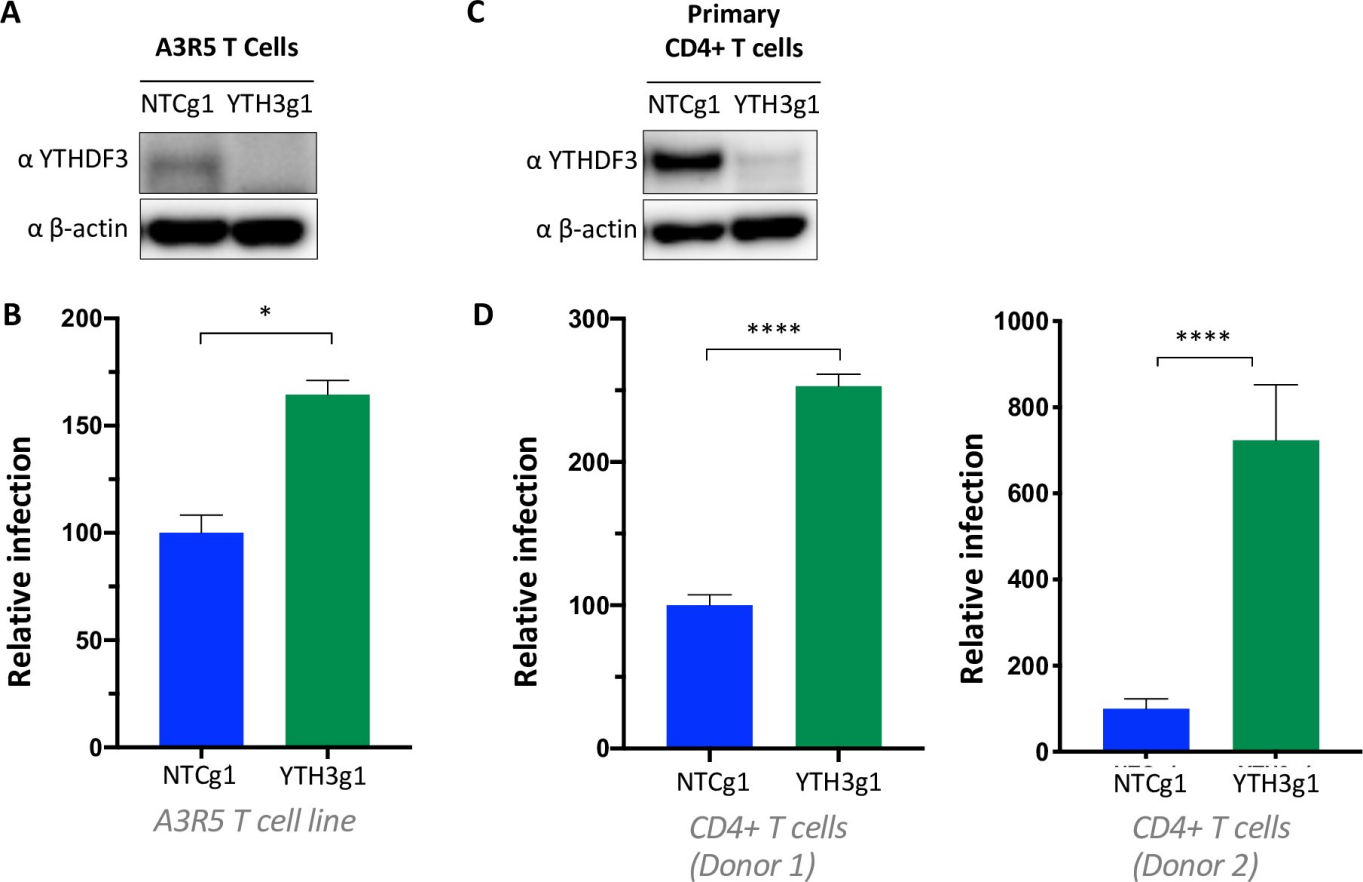
YTHDF3-deficient T cells are more susceptible to HIV infection. **(A)** A3R5-Rev-GFP NTCg1 and A3R5-Rev-GFP YTHDF3g1 T cells were generated by nucleofection of *in vitro* assembled CRISPR/Cas9 ribonucleoproteins. A representative western blot is shown. NTCg1: non targeting guide RNA 1; YTH3g1: YTHDF3 gRNA1. **(B)** Infection of A3R5-Rev-GFP NTCg1 and A3R5-Rev-GFP YTHDF3g1 with HIV NL4-3. The number of infected cells (GFP+) was measured by FACS at day 4 post infection. Infections were performed in duplicates. The number of infected A3R5-Rev-GFP YTHDF3g1 cells (GFP+) is shown relative to the infected A3R5-Rev-GFP NTCg1 cells. * denotes p ≤ 0.05 as determined by an unpaired, two-tailed student’s T test. Three independent experiments were performed. **(C)** YTHDF3 knockdown in primary human CD4+ T cells nucleofected with CRISPR/Cas9 ribonucleoproteins. A representative Western blot is shown. **(D)** Infection of primary CD4+ T cells modified as shown in Fig 3C with HIV-NL4-3 (HIV NL4-3 X4 Renilla Luciferase). Luciferase expression was quantified four days post infection. Infection was calculated relative to NTCg1-targeted T cells. Error bars denote SEM. **** denotes p ≤ 0.0001, as determined by an unpaired, two-tailed student’s T test. Data shown for two different donors (donor 1 and 2) with infection being done in, at least, triplicates.

To determine the role of cellular endogenous YTHDF3 in HIV replication, we generated YTHDF3 knockout A3R5 Rev-GFP T cells by CRISPR-Cas9 nucleofection (**Fig 1A**). Wild type, non-target control (A3R5-Rev-GFP-NTCg1) and A3R5-Rev-GFP-YTHDF3g1 T cells were infected with replication-competent HIV-1 NL4-3 and infection was followed for 3–4 days. We found that A3R5-Rev-GFP-YTHDF3g1 T cells were significantly more susceptible to viral replication compared to T cells treated with the non-targeting control (**Fig 1B**).

We next validated these observations using primary human CD4+ T cells from different donors. We generated YTHDF3 knockout primary CD4+ T cells by CRISPR-Cas9 nucleofection as described previously [22, 23]. Efficient YTHDF3 knockout in primary CD4+ T cells from several donors was confirmed by Western blot (**Fig 1C, S1A Fig**). CD4+ T cells electroporated with YTHDF3 gRNA or NTC gRNA and stimulated with CD3/CD28 were infected in triplicate with replication competent HIV NL4-3 expressing luciferase. Depending on the donor, CD4+ T cells with reduced YTHDF3 expression were between 2.5 and 7-fold more susceptible to infection compared to CD4+ T cells electroporated with the non-targeting control (**Fig 1D**). Similar results were obtained with CCR5-using HIV reporter viruses expressing luciferase, showing that coreceptor usage did not play a role (**S1B Fig)**. Thus, endogenous YTHDF3 protein limits viral replication in both immortalized and primary CD4+ T cells.

### YTHDF3 is incorporated into HIV viral particles in a nucleocapsid-dependent manner

We hypothesized that the m^6^A effector YTHDF3 is encapsidated into egressing HIV particles based on the fact that a global proteomic map for HIV-host interactions showed that the YTHDF3 protein interacts with the HIV-1 nucleocapsid (NC) protein [24].

**Fig 2:**
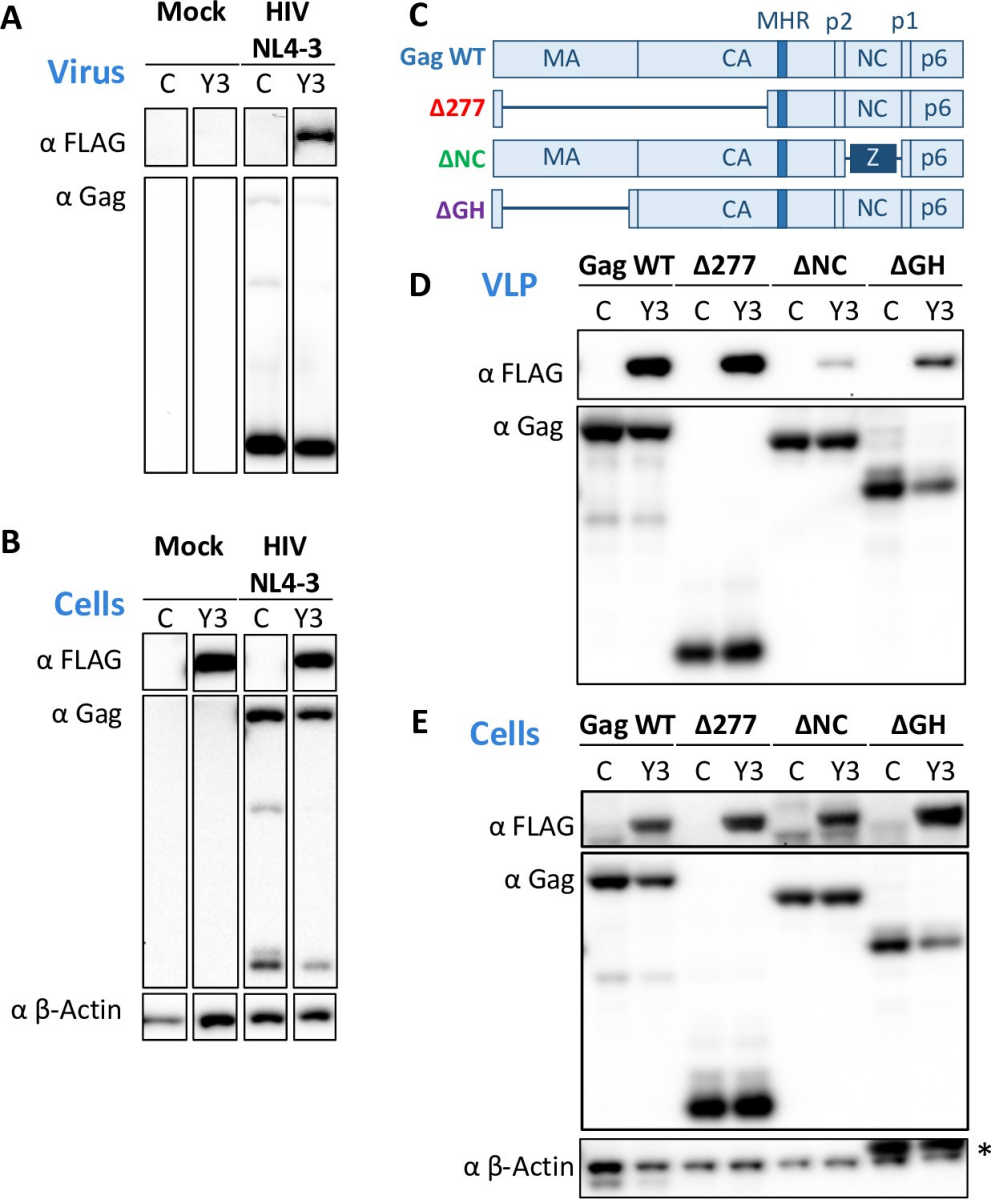
YTHDF3 are incorporated into virions in a nucleocapsid dependent manner. **(A, B)** Concentrated viral supernatants (A) and cell lysates (B) of HEK293T cells co-transfected with HIV NL4-3 and individual plasmids expressing FLAG-tagged YTHDF3 (Y3) or control (C) were analyzed by Western blotting. The antibodies used are denoted. A representative Western blot of five individual experiments is shown. **(C)** Schematic representation of the four HIV NL4-3-derived Gag plasmids used is shown. **(D, E)** Concentrated viral like particles (VLP, D) and cell lysates (E) of HEK293T cells co-transfected with Gag WT or Gag mutant expression plasmids and FLAG-tagged YTHDF3 (Y3) or control (C) plasmids were analyzed by Western blotting. The antibodies used are denoted. A representative Western blot of five individual experiments is shown. * Please note that β-actin control for ΔGH overlaps with GagΔGH.

To directly test this hypothesis, we produced HIV NL4-3 in the presence of FLAG-YTHDF3. Western blotting of concentrated viral supernatants showed that YTHDF3 was present in virions but absent in culture supernatants from mock transfections (**Fig 2A and 2B**). Next we took advantage of an existing panel of HIV NL4-3 Gag expression plasmids used previously to characterize APOBEC3 virion incorporation [25] (see **Fig 2C** for a cartoon illustrating the constructs used) to determine which specific region of HIV Gag is necessary for YTHDF3 incorporation.

We co-transfected these Gag expression plasmids with FLAG-YTHDF3 and analyzed the concentrated viral like particles (VLP) by Western blot. Our results showed that the HIV nucleocapsid protein was necessary for the incorporation of YTHDF3 into VLPs (**Fig 2D and 2E**), and that replacing the HIV nucleocapsid with a leucine zipper failed to mediate YTHDF3 incorporation. Taken together, the m^6^A effector protein YTHDF3 is incorporated into full-length HIV NL4-3 and HIV VLPs through interaction with the Gag nucleocapsid protein.

### YTHDF3 limits viral production and infectivity

**Fig 3:**
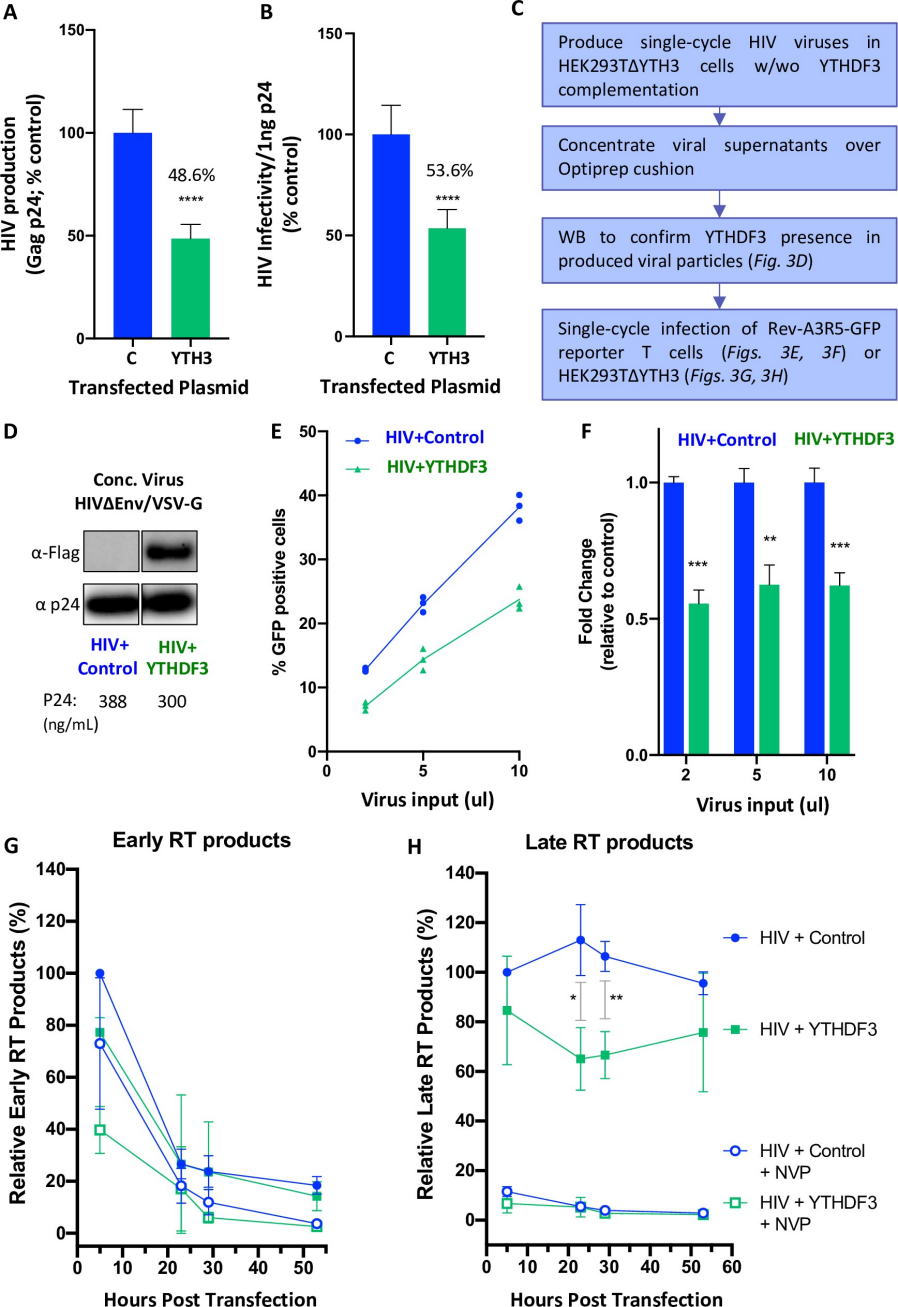
YTHDF3 incorporation in HIV particles negatively affects virus infection. **(A)** HEK293T cells were co-transfected with HIV NL4-3 and plasmids expressing FLAG-tagged YTHDF3 or control. P24 levels in the viral supernatant were measured to quantify virus production. Error bars denote standard error of the mean (SEM). *** denotes p<0.001 and **** denotes p<0.0001 as determined by student’s two-tailed T test. The average of two experiments is shown with each experiment comprising three biological replicates. **(B)** Viral infectivity was determined by infection of TZM-bl cells with 1ng p24 equivalent of each virus produced as shown in Fig 3A. Error bars denote standard error of the mean (SEM). ** denotes p<0.01 and **** denotes p<0.0001 as determined by a student’s two-tailed T test. The average of two experiments is shown with each experiment comprising three biological replicates. **(C)** Experimental approach to directly test the impact of virion-incorporated YTHDF3 on the next round of infection (panels 2C, 2D, 2E). Please note that HEK293TΔYTH3 cells are used for these experiments. **(D)** Single cycle HIV stocks were produced by co-transfecting HEK293TΔYTH3 cells with NL4-3 lacking a functional envelope (NL4-3 ΔEnv), VSV-G envelope, and FLAG-YTHDF3 or control. Culture supernatants were concentrated and analyzed by Western blotting. The viral stocks are referred to as HIV+control (blue) versus HIV+YTHDF3 (green). **(E)** A3R5-Rev-GFP T cells were infected with different doses of single cycle viral stocks (HIV+YTHDF3 versus HIV+control). Quantification of GFP+ expressing T cells was performed by FACS in technical triplicate 3–4 days post infection. One representative experiment with technical triplicates is shown (total number of experiments = 3). **(F)** Normalization of the data shown in panel 3E. Fold change relative to control was calculated. Each row was analyzed individually, without assuming a consistent standard deviation. ** denotes p<0.01 and *** denotes p<0.001 as determined using the Holm-Sidak method, with alpha = 0.05. Data from three individual experiments were included in the graph. **(G)** Infection of HEK293T-ΔYTH3 with HIV+YTHDF3 or HIV+control single cycle viruses in the presence or absence of the non-nucleoside reverse transcriptase inhibitor Nevirapine (NVP, 1mM). Quantification of early RT products was performed using DNA from infected cells harvested at 5, 23, 29, and 53 hours post-infection. Two biological replicates were measured in duplicate or triplicate. Data shown is representative of two independent experiments. Corresponding legends are shown in Fig 3H. **(H)** Same infection as shown in panel 3G only that the quantification of late RT products is shown. Two biological replicates were measured in duplicate or triplicate. * denotes p<0.05 and ** denotes p<0.01 as determined by two-tailed student’s T-test. Data shown is representative of two independent experiments.

We next tested how YTHDF3 incorporation into egressing viruses affects viral infectivity. Since previous reports indicated that YTHDF3 may influence viral production [19], we determined the Gag p24 concentration of the viral stocks produced upon overexpression of YTHDF3. We observed a reproducible 2-fold reduction of viral production upon the expression of FLAG-YTHDF3 protein (**Fig 3A**). To determine infectivity of these viruses, TZM-bl reporter cells were infected with equivalent amounts of Gag p24. We found that viruses which incorporated YTHDF3 were significantly less infectious than viruses produced in the absence of this protein (**Fig 3B**). These results indicate that virion incorporation of YTHDF3 limits HIV infectivity in the next round of infection.

Although YTHDF3 functions upstream of YTHDF1/2 and may therefore regulate their access to RNA [17], its impact on the early steps of the viral life cycle remains to be clearly defined. Here we show that overexpression of YTHDF3 limits viral infectivity in a dose dependent manner in CRISPR/Cas9 YTHDF3-knock out HEK293T cells (293T-ΔYTHDF3, **S2A–S2C Fig**). Next we produced single-cycle viruses in 293T-ΔYTHDF3 cells in the presence of FLAG-YTHDF3 or a control plasmid (**Fig 3B**) to directly determine the effects of virion incorporated YTHDF3 on the next round of infection without the interference of the endogenous YTHDF3 protein. The resulting NL4-3Δenv VSV-G-pseudotyped single cycle viruses were concentrated and termed “HIV+control” or “HIV+YTH3” (**Fig 3C**) depending on whether or not a control plasmid or FLAG-YTHDF3 were co-transfected. A3R5 reporter T cells (Rev-A3R5-GFP) were infected with three different doses of these viral stocks and the number of GFP expressing cells was determined 3–4 days post infection (**Fig 3D)**. YTHDF3 incorporation into virions consistently reduced the number of productively infected CD4+ T cells by approximately two-fold (**Fig 3E and 3F).**

We then determined the step whereupon virus-encapsidated YTHDF3 limits the next round of virus replication. Briefly, 293TΔYTHDF3 cells were infected with the “HIV+control” or “HIV+YTH3” viral stocks and infected cells were collected at 5, 23, 29, and 53 hours post infection (hpi) to quantify early and late HIV reverse transcription products, an indicator of RT activity. Infections in the presence of the RT inhibitor Nevirapine (NVP) served as controls. YTHDF3-containing viruses displayed fewer late RT products than viruses without YTHDF3, while comparable levels of early RT products were observed for both viruses (**Fig 3G and 3H and S3D and S3E Fig**).

Taken together, these results indicate that virion incorporated YTHDF3 alone is sufficient to negatively regulate viral infectivity in the next round of infection by limiting production of late RT products.

### HIV protease cleaves virion incorporated YTHDF3

Since we see an effect on the early steps of the viral life cycle (**Fig 3H**), we next examined whether endogenous YTHDF3 was present in viruses produced by T cells. The detection of endogenous YTHDF3 is complicated by the inconsistent quality of commercially available antibodies. We compared four different YTHDF3 antibodies and two different lots of the same antibody using cell lysates obtained from the A3R5-Rev-GFP-NTCg1 or YTHDF3g1 T cell lines used for the spreading infection experiments (**S3 Fig**). Based on these results, and although there are substantial differences between batches, we selected antibody ab103328 (Abcam), which recognizes an epitope localized between residues 235 and 265.

**Fig 4:**
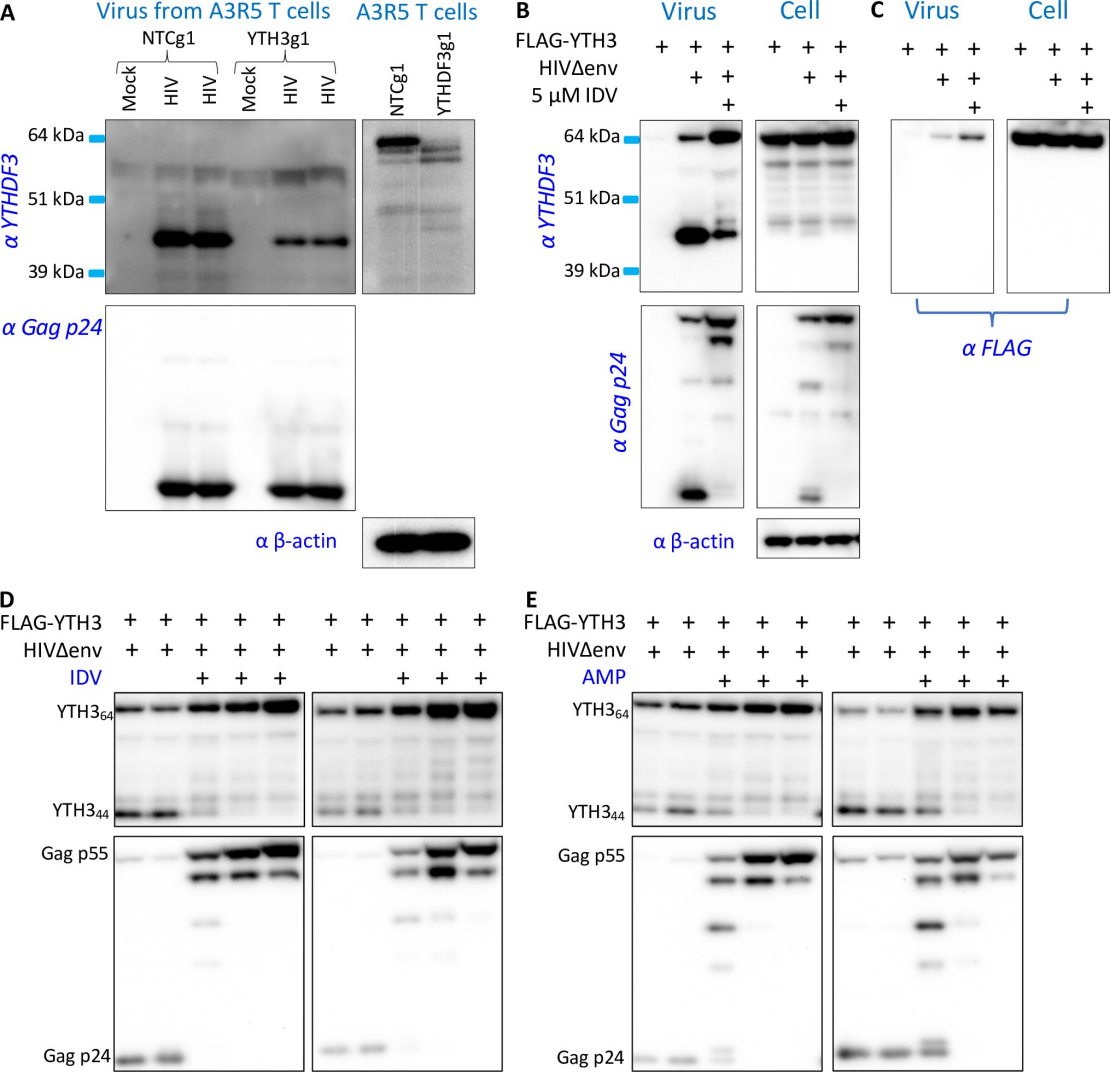
HIV Protease cleaves YTHDF3 in the virus. **(A)** Endogenous YTHDF3 is incorporated into HIV. Western blot of concentrated HIV NL4-3 (left) and A3R5-Rev-GFP NTCg1 or A3R5-Rev-GFP YTHDF3g1 T cell lysates (right) are shown. HIV NL4-3 viral stocks were produced by spreading infection of A3R5-Rev-GFP NTCg1 and A3R5-Rev-GFP YTHDF3g1 T cells in duplicate. Mock represents concentrated culture supernatants collected from mock infected A3R5-Rev-GFP NTCg1 or A3R5-Rev-GFP YTHDF3g1 T cells. Western blots were probed with anti-YTHDF3 ab103328, anti-Gag-p24, and anti-beta-actin. **(B)** HIV NL4-3Δenv virus was produced by co-transfecting HEK293TΔYTH3 cells with FLAG-YTHDF3 in the presence and absence of the protease inhibitor Indinavir (IDV 5 μM). Concentrated virus and cells were lysed and analyzed by Western blotting. Membranes were probed with anti-YTHDF3 ab103328 and anti-Gag-p24. Data is representative of five independent experiments. **(C)** The same samples as shown in Fig 4B were probed with the FLAG epitope antibody used in previous experiments. **(D, E)** NL4-3Δenv virus was produced by co-transfecting HEK293TΔYTH3 cells with FLAG-YTHDF3 in the presence of increasing concentrations of protease inhibitors Indinavir (IDV, range 0–5μM, panel D) or amprenavir (AMP, range 0–5μM, panel E). Western blots of two biological replicates are shown.

We infected A3R5-Rev-GFP-NTCg1 or YTHDF3g1 T cells with NL4-3 and allowed the infection to spread over multiple rounds. At day 12 post infection, culture supernatants were concentrated, HIV p24 content was measured, and comparable amount of p24 viral lysates were analyzed by Western blotting. The virion incorporated, endogenous YTHDF3 protein was detected predominantly as a shorter fragment (around 44 kDa on SDS-PAGE) compared to the full-length 64 kDa long YTHDF3 protein present in the cell lysates of the A3R5 producer cells (**Fig 4A**). Of note, the shorter YTHDF3 band is detected in the concentrated viral lysates produced by the A3R5-Rev-GFP-NTCg1 and to a lesser extent in the A3R5-Rev-GFP-YTHDF3g1 T cells. The latter is likely due to the fact that the A3R5-Rev-GFP-YTHDF3g1 cells represent a population of genome edited cells, which harbor a small amount of wild-type YTHDF3 as demonstrated by the weak 64 kDa YTHDF3 band seen in the YTHDF3g1-targeted T cell lysates (see Fig 4A right panel, and S3 Fig).

A common HIV feature is its ability to neutralize the antiviral activity of host negative regulators. We explored, therefore, whether HIV counters the effect of the host YTHDF3. Since HIV protease is the only known protease inside the virus that could generate such a proteolytic product, we next tested its involvement in the generation of the 44 kDa YTHDF3 fragment. We produced HIV stocks in HEK293T cells in the presence of FLAG-YTHDF3 with and without protease inhibitor treatment (Indinavir, IDV, **Fig 4B**). The 44 kDa YTHDF3 fragment was dramatically reduced in virions in the presence of protease inhibitor treatment, suggesting the cleavage of YTHDF3 is, indeed, HIV protease-dependent (**Fig 4B**). The 44 kDa fragment was not detectable in the producer cell lysates, which is in line with the fact that HIV protease is only present in the mature viral particle and not in the producer cells. Importantly, the shorter YTHDF3 fragment was not detected when the FLAG epitope tag antibody was used to probe the concentrated viruses (compare “Virus” in **Fig 4B** with “Virus” in **Fig 4C**) indicating the likely instability of the Flag-tagged N-terminally processed fragment.

We next performed experiments to determine whether YTHDF3 proteolytic processing within the virion could be blocked in a dose dependent manner using two different FDA-approved protease inhibitors (Indinavir, Amprenavir). As predicted, the amount of the full-length YTHDF3 increased while the 44 kDa YTHDF3 form decreased to undetectable levels in the virions produced in the presence of increasing concentrations of Indinavir or Amprenavir (**Fig 4D and 4E**). YTHDF3 cleavage in concentrated virions closely mirrored the HIV Gag p55 processing, that also was inhibited in a dose-dependent manner by both protease inhibitors (see lower panels of **Fig 4D** and **4E**).

**Fig 5:**
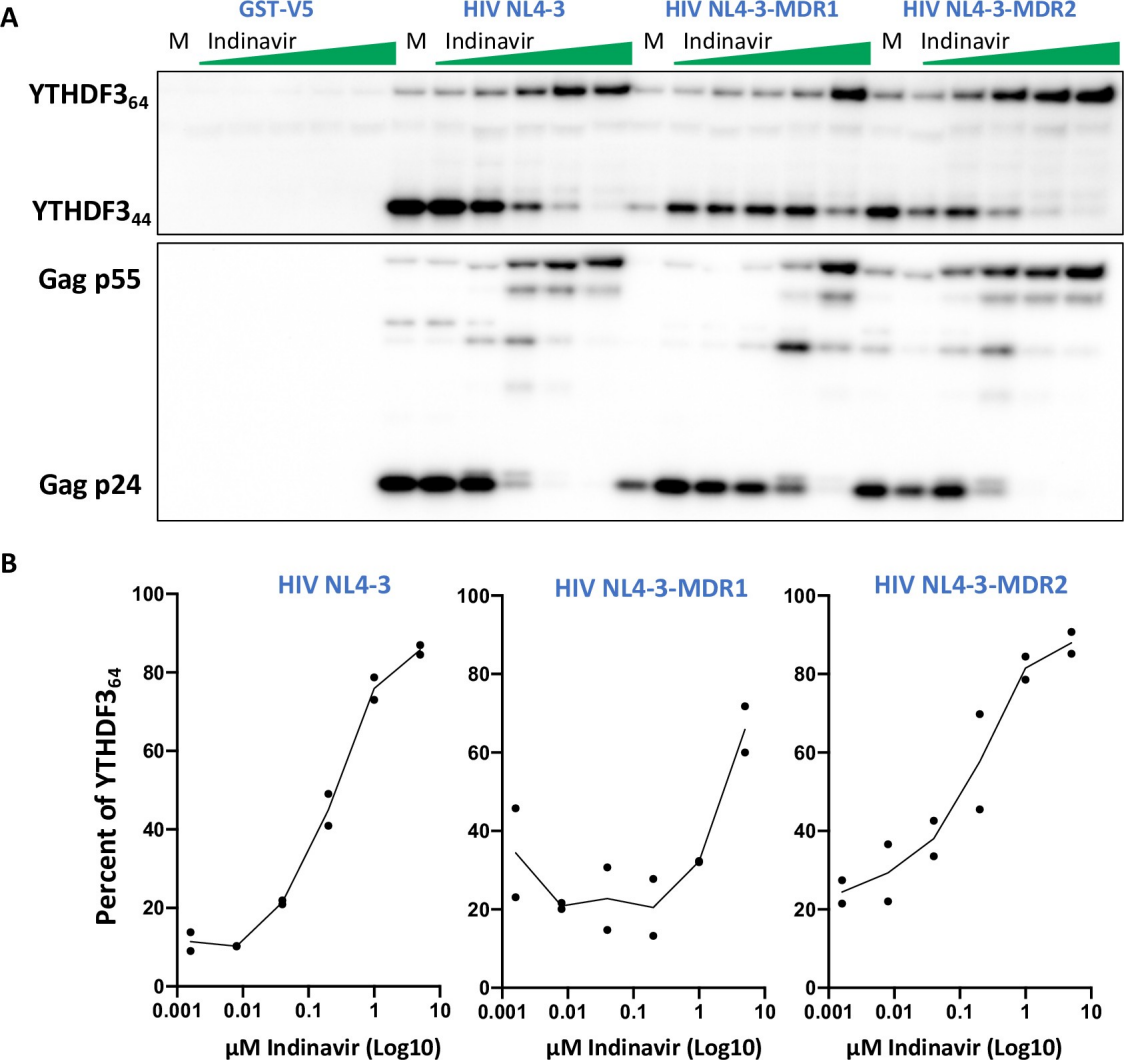
Cleavage ofYTHDF3 in protease-resistant HIV mutant strains. **(A)** Western blot of concentrated virus lysates produced in HEK293TΔYTH3 cells co-transfected with FLAG-YTHDF3 and HIV NL4-3Δenv WT, NL4-3Δenv-MDR1 (Indinavir resistance associated mutations I84V & L90M), NL4-3Δenv-MDR2 (L90M), or control plasmid. Increasing doses of Indinavir or DMSO-only control was added to cells one day after transfection. Membranes were probed with anti-YTHDF3 ab103328 and anti-Gag p24. Data shown is representative of three individual experiments. **(B)** Quantification of virion incorporated full-length YTHDF3 protein (64 kD, YTHDF3_64_) and the YTH3 fragment (44 kD, YTHDF3_44_) as determined by Western blot (e.g., as shown in Fig 5A). Percent YTHDF3_64_ was calculated relative to the total YTHDF3_64_ and YTHDF3_44_ in each lane. Data shown for two independent experiments are shown. The DMSO-only control is artificially placed at 0.0016 μM for the purpose of graphical representation.

We next tested whether viral variants with reduced susceptibility to protease inhibitors displayed altered YTHDF3 proteolytic cleavage efficiency. Isogenic NL4-3 ΔEnv EGFP viruses encoding protease and RT regions from multi-drug resistant HIV (see Table 1 for a summary of the drug resistance associated mutations) were produced in the presence and absence of FLAG-YTHDF3, treated with increasing concentration of Indinavir, concentrated and analyzed by Western blot. Briefly, HIV-MDR1 encodes two major protease inhibitor resistance associated mutations (PR I84V and L90M) while HIV-MDR2 only has one (PR L90M). As expected, wild-type HIV NL4-3 virions displayed a protease inhibitor dose dependent increase of full length YTHDF3 (**Fig 5A**). In contrast, protease resistant HIV viruses allowed for HIV Gag and YTHDF3 processing at the IDV concentrations that inhibited the processing in the wild-type HIV NL4-3 (**Fig 5A**). Moreover, YTHDF3 processing in concentrated HIV-MDR1 viruses could only be partially blocked at the highest protease inhibitor concentration tested while HIV wild-type and HIV-MDR2 viruses incorporated only full-length YTHDF3 at the higher Indinavir concentrations (**Fig 5B**).

Thus, endogenous and ectopically expressed YTHDF3 incorporated into HIV virions is predominantly proteolytically processed with most of the virion-incorporated YTHDF3 being the short 44 kDa fragment. Importantly, this process only occurs in the virion and is fully inhibited when viral stocks are produced in the presence of HIV protease inhibitors.

**Table 1:** Overview of the drug resistance associated mutations found in the protease and RT regions of HIV NL4-3-MDR1 and HIV NL4-3-MDR2. The molecular backbone is isogenic (HIV NL4-3 Δenv/eGFP). The protease mutations associated with reduced susceptibility to Indinavir and Amprenavir are in bold.

		HIV NL4-3-MDR1	HIV NL4-3-MDR2
**Protease**	**Protease Resistance Mutations**	L10V, I13V, G16A, K20R, E35D, M36I, R41K, I64V, H69K, A71V, **I84V**, **L90M**, I93M	I13V, I64V, V77I, **L90M**
**Reverse Transcriptase**	**NRTI Resistance Mutations**	M41L, L74V	M41L
	**NNRTI Resistance Mutations**	L100I, K103N	V106A
	**Other Mutations**	E6K, V35K, K49R, I50V, K122E, D123E, I135L	K43E, S68G, K122E, D123E, I135T

### HIV protease cleaves virion incorporated YTHDF3 at two or more different sites

**Fig 6:**
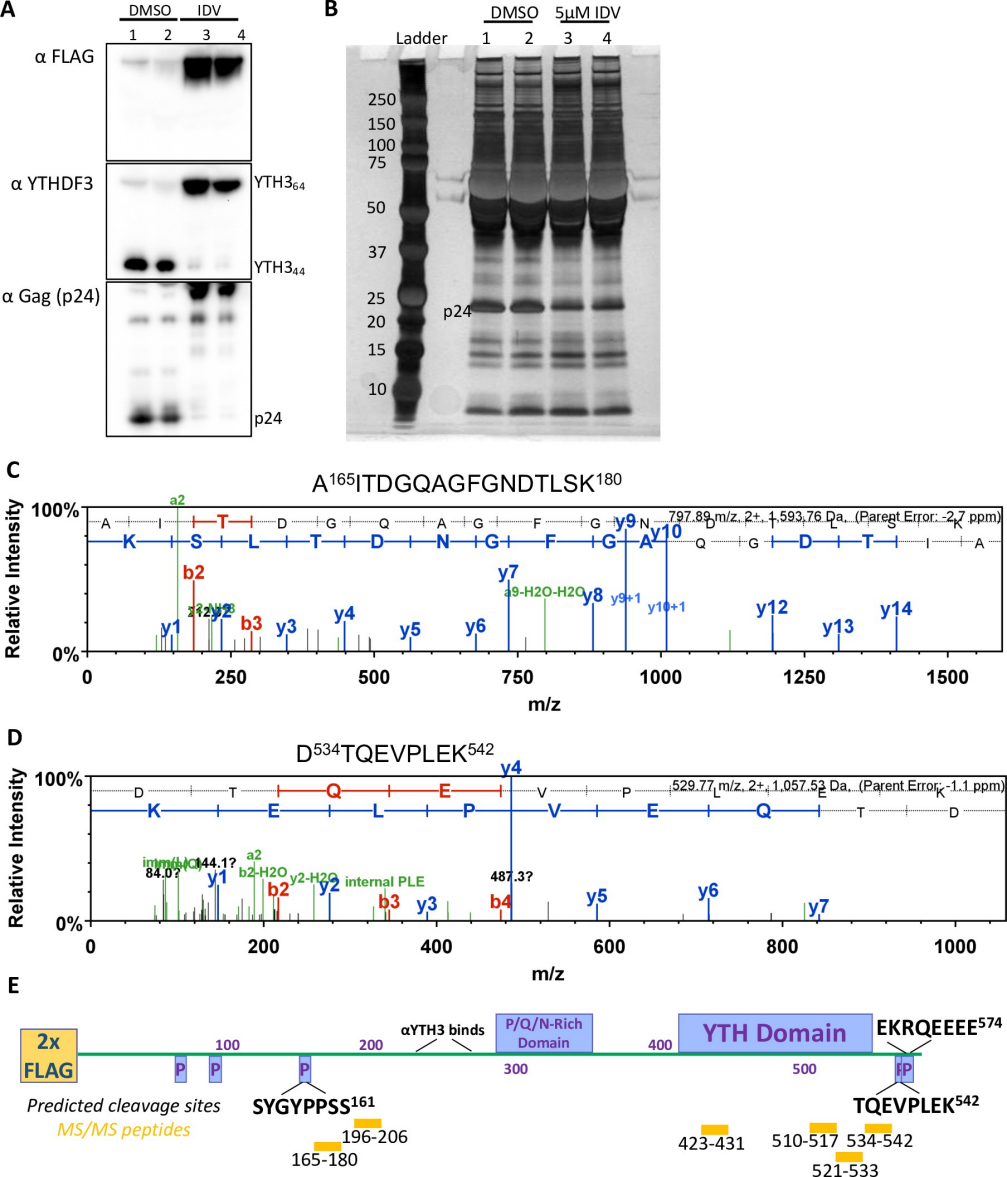
Detection and mapping of YTHDF3 fragments by LC/MS/MS. **(A)** Western blot of concentrated viruses produced by co-transfection of HEK293TΔYTHDF3 with HIV NL4-3 and FLAG-YTHDF3 in the presence or absence of Indinavir (IDV, 5μM). **(B)** Silver stain SDS PAGE gel of the viral stocks shown in Fig 6A. The region of interest was cut out and sent for LC/MS/MS. **(C)** The most N-terminal peptide identified by mass spectrometry is shown (peptide sequence is shown above the spectrum). **(D)** The most C-terminal peptide identified by mass spectrometry is shown (peptide sequence is shown above the spectrum). **(E)** Graphical representation of the different protein domains within YTHDF3, the unique tryptic peptides identified by mass spectrometry (MS/MS peptides, yellow), the predicted protease cleavage sites (P, black), and the location of the YTHDF3 antibody ab103328 (“αYTH3 binds” box) epitope are shown.

In order to validate our previous findings, we decided to identify and map the YTHDF3 fragments by LC/MS/MS incorporated into HIV virions. Briefly, HIV viruses were generated in duplicate in the presence of FLAG-YTHDF3 and Indinavir, concentrated and separated on SDS-PAGE. In addition to Western blotting (**Fig 6A**), we performed a silver stain of the gel and excised the bands running around 40 kDa (**Fig 6B**). LC/MS/MS identified five unique tryptic peptides (**Fig 6C and 6D**), which mapped to the region between amino acids 165 and 542, which comprises the P/G/N-rich domain and the YTH domain of YTHDF3 (**Fig 6E**).

Next we used a protease cleavage site prediction software (HIVcleave [26, 27]) to identify potential HIV protease cleavage sites in the full-length YTHDF3 protein. The five sites with the highest confidence are highlighted in the YTHDF3 cartoon (**Fig 6E**) along with the epitope sites of the FLAG and YTHDF antibodies used. Based on these predictions, it is likely that the full-length YTHDF3 protein is cleaved by HIV protease at SYGY|PPSS (residues 154–161) and either TQEV|PLEK (residues 535–542) or EKRQ|EEEE (residues 567–574), yielding a product of about 380–412 amino acids (about 42–45 kDa predicted size). Taken together, our results confirm the identity of the short YTHDF3 fragment and indicate that HIV protease cleaves the virus-incorporated YTHDF3 protein at two or more sites.

## Discussion

In the context of HIV replication, RNA modifications can influence HIV cDNA synthesis (RNA→DNA; *specific for HIV*), HIV gene transcription (RNA→Protein, same as for cellular genes), and HIV protein translation and assembly (HIV RNA:protein interactions). In this study we focus on dissecting the interplay between HIV and m^6^A effector protein YTHDF3 during the early steps of the HIV life cycle.

We observed that endogenous YTHDF3 expression limits the cell’s susceptibility to HIV infection, since human CD4+ T cells with CRISPR-Cas9 edited YTHDF3 locus (no detectable YTHDF3 protein expression) better support HIV infection (see Fig 1). These findings are in good agreement with reports showing that viruses produced in YTHDF3-deficient cells are more infectious than virus produced in wild-type cells [19].

To date, no studies have looked specifically at the impact of virion-incorporated m^6^A effector proteins based on the assumption that these proteins are absent from egressing viral particles [9, 11, 12, 19]. We present clear evidence that YTHDF3 is incorporated into Gag viral like particles in a nucleocapsid dependent manner (**Fig 2**) as well as in viruses produced by T cells (**Fig 4**). Our observations are in line with previous reports showing a direct interaction between YTHDF3 and nucleocapsid [24]. Since the interaction of YTHDF3 with HIV Gag p55 was reported to be RNA dependent [19], YTHDF3 may exploit Gag nucleocapsid-mediated RNA recruitment to gain access to the virion in a manner reminiscent of how APOBEC3G is incorporated into viral particles [25].

We demonstrate that viruses produced in cells overexpressing YTHDF3 are less infectious than virus produced in wild-type cells after adjusting for the reduction in virus production (**Fig 3**). These observations suggest that the presence of YTHDF3 in the producer cells regulates viral infectivity in the next round of infection. By producing single cycle viruses in YTHDF3 deficient cells with and without YTHDF3 complementation, we show that virion-incorporated YTHDF3 is sufficient to limit infection of CD4+ T cells by acting at the step of reverse transcription (**Fig 3**). Since reverse transcription starts in the mature virion prior to fusion with the cellular plasma membrane [28–30], virus-incorporated YTHDF3 is optimally positioned to interfere with this early step of the viral life cycle by binding to m^6^A modified sites on the HIV RNA genome.

The explanation for why YTHDF3 incorporation into HIV particles has not been appreciated until now is simple. HIV protease cleaves YTHDF3 incorporated into the virus resulting in the release, and likely degradation, of the amino-terminal FLAG tag (**Figs 4 and 5**). Probing concentrated virions with antibodies recognizing the amino-terminal tag rather than the central region of the YTHDF3 protein directly, thus, leads to false negative results. Similarly, virion incorporated endogenous YTHDF3 is only present in the shorter, 44 kDa form given that it is completely processed by HIV protease (**Fig 4C**). Thus, mature HIV particles harbor a previously unknown, unique version of this m^6^A reader protein rather than the full-size, wild-type version of the YTHDF3 (64 kDa) found in the producer cells.

Treatment of HIV producer cells with different FDA approved protease inhibitors (e.g., Indinavir, Amprenavir) prevented cleavage of YTHDF3 resulting in a large increase of the full-length YTHDF3 protein relative to the cleaved YTHDF3 product (**Fig 4**). The accumulation of this shorter YTHDF3 fragment in virions produced in wild-type cells suggests that HIV protease processes most of the endogenous YTHDF3 under these near physiological conditions (**Fig 4**). HIV protease is only present in the mature HIV particle and, thus, it is not surprising that the short 44 kDa YTHDF3 fragment is not detectable in producer cell lysates (**Fig 4B, right**). Unfortunately, we cannot yet test the effect of blocking HIV protease cleavage of YTHDF3 on the viral life cycle as the addition of the protease inhibitor prevents Gag processing and automatically renders the virus non-infectious. Mutating the potential cleavage site(s) in the YTHDF3 protein can also be problematic as such mutants can have unintended consequences for the cellular YTHDF3 function.

A hallmark of a HIV restriction factor is the fact that HIV has evolved to counteracting it. It was previously reported that the anti-HIV protein APOBEC3H SV200 is cleaved by the HIV protease resulting in an attenuated function of the cleaved APOBEC3H SV200 variant [31]. HIV protease processing of YTHDF3 may, thus, be the mechanism by which HIV counteracts the antiviral activity of YTHDF3. If true, it is likely that our results described here underestimate the antiviral effects of endogenous, full-length YTHDF3 given the viral antagonism. Our results establish the m^6^A effector YTHDF3 as a restriction factor acting on the early steps of the viral life cycle if left unchecked by the HIV protease.

It is attractive to speculate that the extended antiviral activities of protease inhibitors are, in part, derived from the restoration of the antiviral activity of m^6^A reader proteins such as YTHDF3. Viral hypersusceptibility to protease inhibitors as well as mutations in Gag that modulate the amount of mature protease produced have been described [32, 33]. Future studies are needed to determine the true antiviral potency and breadth of the YTHDF3 protein when combined with protease inhibitors.

## Materials and methods

### Plasmids

The following reagents were generous gifts from colleagues: N-terminal FLAG-tagged YTHDF3 proteins cloned into pEFTak (Dr. S. Horner, Duke University [12]); pcrVI NL4-3 Gag plasmids (Dr. P. Bieniasz, The Rockefeller University [25]), replication-competent HIV-1 NL4-3 expressing CCR5 or CXCR4 using envelopes as well as the luciferase reporter (Dr. F. Kirchhoff, University of Ulm). HIV NL4-3 Δenv-eGFP (reagent #11100 [34]) and NL4-3 (reagent #114 [35]) were obtained through the AIDS Research and Reference Reagent Program, Division of AIDS, NIAID, National Institutes of Health.

Insertion of the resistance-conferring regions into the HIV NL4-3- Δenv-eGFP based molecular background were performed as previously described [36]. NL4-3-Δenv-eGFP MDR1 and MDR2 encode the following drug resistance associated mutations (MDR1 Protease: L10V, I13V, G16A, K20R, E35D, M36I, R41K, I64V, H69K, A71V, I84V, L90M, I93M; MDR1 RT: E6K, V35K, M41L, K49R, I50V, L74V, L100I, K103N, K122E, D123E, I135L; MDR2 Protease: I13V, I64V, V77I, L90M; MDR2 RT: M41L, K43E, S68G, V106A, K122E, D123E, I135T, see also Table 1).

### Cell culture

Human embryonic kidney HEK293T cells (ATCC) and TZM-bl cells (cat# 8129, NIH AIDS Reagent Program, Division of AIDS, NIAID, National Institutes of Health) were cultured in Dulbecco's modified Eagle medium (DMEM) in the presence of 10% fetal bovine serum (FBS; GemCell), 100 IU penicillin, and 100 μg/mL streptomycin at 37°C and 5% CO_2_.

ViroVision Rev-A3R5-GFP/Luc HIV Reporter Cells (Cube BioSystems) were cultured in RPMI containing 10% fetal bovine serum (FBS; Gibco), 100 IU penicillin, and 100 μg/mL streptomycin at 37°C and 5% CO_2_. All cell lines were mycoplasma free as confirmed by regular testing using the MycoAlert Mycoplasma Detection kit (Lonza).

Primary human CD4+ T cells were purified from peripheral blood lymphocytes obtained from anonymous healthy blood donors (New York Blood Center). Ficoll (Ficoll Hystopaque; Sigma) density centrifugation was performed as per the manufacturer’s instructions, and CD4+ T cells were negatively selected using magnetic beads (CD4+ T-cell isolation kit I; Miltenyi Biotec). Isolated CD4+ cells were maintained in RPMI 1640 supplemented with 10% FBS (Gibco), 100 IU penicillin, 100 μg/mL streptomycin, 2 mM L-glutamine, and 20 U/mL recombinant human IL-2 (NIH AIDS Reagent Program, Division of AIDS, NIAID, National Institutes of Health).

### Ethics statement

Peripheral blood lymphocytes were purchased from New York Blood Center from anonymous donors. The investigators had no direct interactions with blood donors or influence on the selection of PBMCs. This work is regarded as non-human subject research.

### Genome editing

Primary human CD4+ T cell crRNP-mediated gene editing experiments were carried out as previously described [22, 23]. Nucleofection was performed using the Amaxa P3 Primary Cell Nucleofector Kit (Lonza). Briefly, primary CD4+ T cells were stimulated for 3 days at 500 μL per well (2.5e6 cells/mL) stimulated with plate-bound anti-CD3 (Clone UCHT1; Tonbo) and suspended anti-CD28 (5 μg/mL; Clone CD28.2; Tonbo). Cas9 RNPs were prepared up to 24 hours before each experiment. Guide RNA (gRNA) was designed specifically to target YTHDF3 (YTH3g1: insert sequence) using the Benchling tool. The non-targeting control guide (NTCg1) used was previously published [23].

gRNAs, tracrRNA and Cas9-3NLS HiFi protein were purchased from IDT. To generate crRNA:tracrRNA duplexes, crRNA and tracrRNA (100 uM each in Duplex Buffer) were combined 1:1 (1.6 μL) and incubated for 30 minutes at 37°C. To generate Cas9 ribonuclein proteins (RNPs), Cas9-3NLS HiFi (1.3 μL) was added to the crRNA:tracrRNA duplexes and incubated for 15 minutes at 37°C. Lastly, electroporation enhancer (IDT) (0.8 μL) was added to the Cas9 RNPs for a final volume of 5.3 μL. The Cas9 RNPs were stored at 4°C until immediately before use. For each nucleofection reaction, one million primary CD4^+^ T cells were resuspended in 20 μL P3 buffer and combined with 5.3 μL Cas9 RNPs. Cells were electroporated using program EH-115 on the Amaxa 4D-Nucleofector X unit (Lonza). 80 μL pre-warmed complete RPMI was added to each well and the cells recovered for a minimum of 30 minutes before transfer to a 96 well plate and cells were re-stimulated with 2 μL Human T-activator CD3/CD28 Dynabeads (Gibco) in a total volume of 200 μL per well.

293TΔYTH3 cells were generated with the Amaxa SF Cell Line Nucleofector Kit (Lonza) and Alt-R CRISPR-Cas9 System (IDT) as described with modifications [37]. Cas9 RNPs were prepared up to 24 hours before each experiment. To generate crRNA:tracrRNA duplexes, crRNA and tracrRNA were combined 1:1 (1.2 μL) and incubated for 30 minutes at 37°C. To generate Cas9 RNPs, Cas9-3NLS HiFi (IDT) (1.3 μL) and Duplex Buffer (0.9 μL) was added to the crRNA:tracrRNA duplexes and incubated for 15 minutes at 37°C. Lastly, electroporation enhancer (IDT) (0.8 μL) was added to the Cas9 RNPs for a final volume of 6 μL. The Cas9 RNPs were stored at 4°C until immediately before use. Approximately 300,000 cells were resuspended in 20uL SF solution and combined with 6 μL Cas9 RNPs. Cells were electroporated using program CM:130 on the Amaxa 4D-Nucleofector X unit (Lonza). 100 μL pre-warmed complete DMEM was added to each well and the cells were transferred to a 24-well plate. A second CRISPR nucleofection was performed to improve knockout efficiency in the cell population.

A3R5-Rev-GFP-YTHDF3g1 cells were generated with the Amaxa SG Cell Line Nucleofector Kit (Lonza) and Alt-R CRISPR-Cas9 System (IDT). Cas9 RNPs were prepared as previously described above for the HEK293T cell nucleofection. 0.5x10^6^ A3R5-Rev-GFP cells were resuspended in 20 μL SG solution and combined with 6 μL Cas9 RNPs. Cells were electroporated using program CM:137 on the Amaxa 4D-Nucleofector X unit (Lonza). 100 μL pre-warmed complete DMEM was added to each well and the cells were transferred to a 48-well plate. Five days post nucleofection, dead cells were removed using the Annexin V Kit (EasySep).

### Transfection experiments

Production of VLPs or viruses to investigate viral incorporation: VLPs or viruses were generated by transfection of HEK293T or HEK293TΔYTH3 cells (as indicated) in 24-well format with 500ng HIV-1 NL4-3 plasmid, NL4-3-derived Gag plasmids, or GST-V5 control plasmid and cotransfected with 500ng YTHDF1-3 plasmids (as indicated). Three days after transfection, culture supernatants were collected, clarified at 500 x g for 10 minutes, and viral particles were purified on a 6% Optiprep cushion (Sigma) by centrifugation at 21,000 x g for 5 hours. Concentrated viral particles were lysed in 1% SDS.

Production of viruses to investigate infectivity: 293T or 293TΔYTH3 cells (as indicated) in a 96-well format were transfected with 100 ng HIV-1 NL4-3 plasmid, or GST-V5 plasmid and cotransfected with 100ng YTHDF1-3 plasmids (as indicated). 100 μL complete DMEM was supplemented the next morning. Three days post transfection, viral supernatants were harvested and used to quantify Gag p24 using HIV-1 p24 ELISA (XpressBio # XB-1000) following manufacturer's instructions and determine viral infectivity.

Replication-competent HIV NL4-3 X4 and R5 Renilla luciferase reporter viral stocks were generated by transfection of HEK293T cells with polyethylenimie (PEI, Polysciences). Three days after transfection, culture supernatants were collected, clarified at 500 x g for 10 minutes and filtered (0.45 μM). Viruses were purified on a 6% Optiprep cushion (Sigma) by centrifugation at 14,000 x g for 6 hours. Viral titers were determined by infecting TZM-bl reporter cells with triplicate serial dilutions of the viral stocks as previously described [38].

### Measurement of viral infectivity

TZM-bl reporter cell-line (cat# 8129, NIH AIDS Reagent Program, Division of AIDS, NIAID, National Institutes of Health[39–43]) harboring the β-galactosidase reporter gene driven by the HIV-1 long terminal repeat, was used to assess the infectivity of NL4-3 viruses produced in presence/absence of YTHDF3 protein expression. TZM-bl cells were infected with either equal volume of viral supernatants or 1ng of Gag-p24 equivalents (as determined by ELISA) of each virus. β-galactosidase activity was quantified two to three days post-infection using the chemiluminescent substrate Tropix, Galacto-Star (Applied Biosystems, Thermo Fisher Corporation). Chemiluminescence was measured on a Victor3 Plate Reader (PerkinElmer).

### Western blots

Cells were lysed two days after transfection using 150 μl of 1% SDS. Primary CD4+ T cells and Rev-A3R5-GFP cells were lysed in RIPA buffer supplemented with complete protease inhibitor (Roche). Viral and cell lysates were run on 10% polyacrylamide gels (Invitrogen, Thermo Fisher Corporation) and transferred to polyvinylidene difluoride (PVDF) membranes (Pierce, Thermo Fisher Corporation). Blots were blocked in 5% milk in 0.1% PBS Tween (PBST) and washed in 0.1% PBST. Western blot chemiluminescence was detected with SuperSignal^TM^ West Femto Substrate (Thermo Scientific). Imaging and quantification of the Western blot bands was performed using AlphaView software (ProteinSimple).

The following antibodies were used for immunoblots: Anti-β-Actin (13E5, 1:1500, Cell Signaling Technology Cat #4970, RRID:AB_2223172); Anti-FLAG (clone M2, 1:750, Sigma-Aldrich F1804, RRID:AB_262044); Anti-YTHDF3 (1:200, Abcam ab103328, RRID:AB_10710895), Anti-YTHDF3 (1:1000 Abcam ab220161), Anti-YTHDF3 (1:1000 Proteintech 25537-1-AP), Anti-YTHDF3 (F-2 1:200 Santa Cruz Biotech sc-377119), and Anti-HIV-1 p24 (1:750, 183–H12-5C, Cat # 1513, NIH AIDS Reagent Program [44]).

### Infection experiments

CD4+ T cell infections: Three days after CRISPR nucleofection, 250,000 primary CD4+ T cells were infected with NL4-3 X4 (CXCR4 using) and NL4-3 R5 (CCR5 using) Renilla Luciferase overnight in the presence of polybrene (1 μg/mL). Cells were washed the next morning and resuspended in R10-IL2 culture media. The level of infection was determined by quantifying Renilla Luciferase four days post infection using the Renilla Luciferase Assay System kit (Promega).

A3R5-GFP reporter infections: 250,000 cells were infected with VSV-G-pseudotyped single-cycle NL4-3ΔEnv viruses overnight in the presence of polybrene (2 μg/mL). Cultures were replenished with fresh media on the next morning. Level of infection was determined by quantification of GFP+ cells by flow cytometry with a Guava EasyCyte Flow Cytometer (Millipore) 3–4 days post infection. An average of 5,000 cells were acquired per sample, and data were analyzed using InCyte (Millipore) software.

A3R5-Rev-GFP NTCg1 or YTHDF3g1 reporter infection to produce viruses: 20 million A3R5-Rev-GFP NTCg1 or A3R5-Rev-GFP YTHDF3g1 cells were infected with HIV NL4-3 in duplicate overnight. On day 1 cell media was changed. 12 days post infection, culture supernatants were clarified by syringe filtration, and viral particles were purified on a 6% Optiprep cushion (Sigma) by centrifugation at 14,000 x g for 5 hours. Concentrated viral particles were lysed in 1% SDS.

HEK293TΔYTHDF3 cell infections to quantify HIV RT transcripts: HIV+Control and HIV+YTHDF3 viral stocks were treated for 1 h at room temperature with DnaseI (40 u/mL; BioLabs) before infection to remove background coming from plasmid DNA. 150,000 HEK293T KO YTHDF3 cells were plated per well in a 24-well plate the day before infection. Cells were pre-treated with polybrene (8 μg/mL) and Nevirapine (NVP) 1 hour before infection. Cells were infected with VSV-G-pseudotyped single-cycle NL4-3ΔEnv viruses in triplicates for 5 hours, upon which cells were washed with PBS and culture media was replenished. HIV-1 early RT products [19], HIV late RT products [45, 46], and unspliced GAPDH [9] were measured at 5, 23, 29, and 53 hours post infection by SYBR green qPCR (Roche). Early RT and late RT product levels were quantified in duplicates relative to unspliced GAPDH levels using the delta cycle threshold (CT) method.

### Silver stain and protein identification mass spectrometry

The lysate of concentrated NL4-3 viruses treated with DMSO or Indinavir (~13 μl) was mixed with 5 μl 4× LDS sample buffer (ThermoFisher, NP0007) and 2 μl 10× sample reducing agent (ThermoFisher, NP0004). The entire mixture (20 μl) was heated at 95°C for 5 minutes and loaded on a 4–12% Bis-Tris denaturing protein gel, together with protein molecular weight standards (Bio-Rad, 1610374). The gel was stained with Pierce silver stain kit (ThermoFisher, 24612) by following the manufacturer’s instructions. Protein bands around the target fragment band region were excised from the gel using the protein molecular weight standard lane as a guide. The gel bands were sent to the Proteomics & Metabolomics Facility at the Center for Biotechnology/University of Nebraska–Lincoln for protein identification mass spectrometry. Mascot (Matrix Science, London, UK; version 2.6.1) was used to analyze all MS/MS samples. MS/MS based peptide and protein identifications were further analyzed by Scaffold (version Scaffold_4.8.4, Proteome Software Inc., Portland, OR).

## Supporting information

S1 Fig HIV infection is increased in primary human CD4+ T cells lacking YTHDF3 expression. **(A)** YTHDF3 knock down/knock out efficiency in primary human CD4+ T cells is assessed by Western blotting. One guide RNA directed against YTHDF3 and one non-targeting guide RNA were evaluated in primary human CD4+ T cells from two different donors. Anti YTHDF3 ab103328 at a dilution of 1/200 was used. Western blots from two different donors are shown. **(B)** Infection of primary human CD4+ T cells with CCR5 using HIV (HIV NL4-3 R5 Renilla Luciferase) was performed in triplicate. Luciferase expression was quantified four days post infection. Infection of YTHDF3g1-targeted T cells was calculated relative to NTCg1-targeted T cells. Error bars denote SEM. ** denotes p ≤ 0.01, as determined by an unpaired, two-tailed student’s T test.
(TIF)

S2 Fig YTHDF3 reduces viral infectivity. **(A)** Western blot of HEK293T-ΔYTHDF3 with anti-YTHDF3 ab103328 and anti-beta actin. YTHDF3 was knocked out using a CRISPR-Cas9 genome editing approach. **(B)** YTHDF3 expression negatively regulates HIV infectivity. Transfections were performed in HEK293T-ΔYTHDF3 cells with increasing amounts of FLAG-YTHDF3 plasmid (25–575 ng) in biological triplicates. TZM-bl reporter cells were infected with 5ul of viral supernatant. Data shown is representative of two independent experiments. **(C)** Western blot of the HEK293T-ΔYTHDF3 producer cells from which the viruses shown in S2C Fig were collected. Membranes were probed with anti-YTHDF3 ab103328, anti-p24 (Gag) and anti-beta actin. The Western blot is representative of two independent experiments.
(TIF)

S3 Fig Detection of endogenous YTHDF3 in A3R5-Rev-GFP NTCg1 and A3R5-Rev-GFP YTHDF3g1 T cells. A panel of four different commercially available YTHDF3 antibodies and two different lots of the same YTHDF3 antibody were used to probe for endogenous YTHDF3 using cell lysates from A3R5-Rev-GFP NTCg1 and A3R5-Rev-GFP YTHDF3g1 T cells. Cellular YTHDF3 is detected at 64 kDa. Anti-beta actin was used as a loading control.
(TIF)
