# Supplementary figures and images for: HIV protease cleaves the antiviral m6A reader protein YTHDF3 in the viral particle
Source: PLoS Pathog. 2020 Feb 13;16(2):e1008305. doi: 10.1371/journal.ppat.1008305 (PMC7043784; doi:10.1371/journal.ppat.1008305)

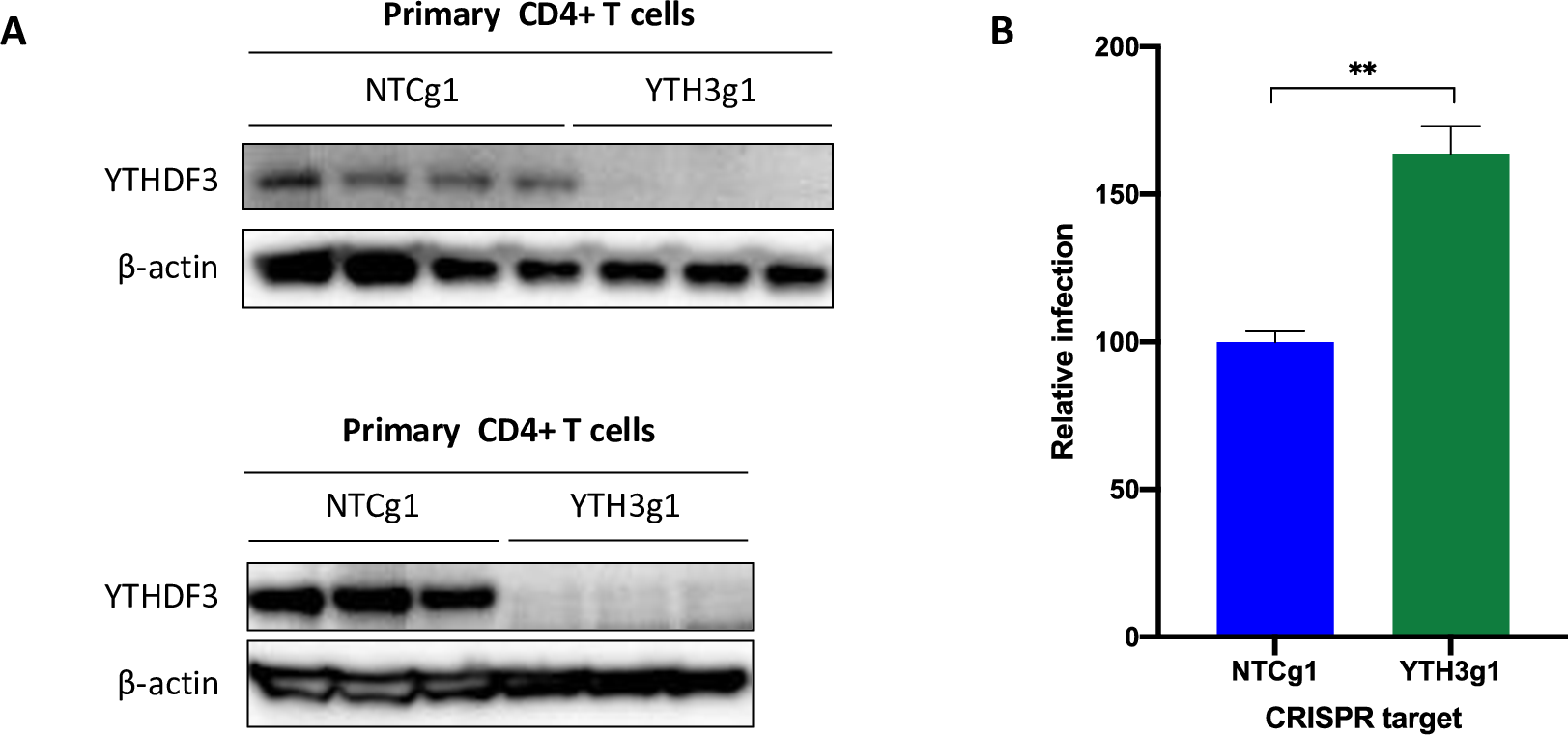

Supplement: S1 Fig — (A) YTHDF3 knock down/knock out efficiency in primary human CD4+ T cells is assessed by Western blotting. One guide RNA directed against YTHDF3 and one non-targeting guide RNA were evaluated in primary human CD4+ T cells from two different donors. Anti YTHDF3 ab103328 at a dilution of 1/200 was used. Western blots from two different donors are shown. (B) Infection of primary human CD4+ T cells with CCR5 using HIV (HIV NL4-3 R5 Renilla Luciferase) was performed in triplicate. Luciferase expression was quantified four days post infection. Infection of YTHDF3g1-targeted T cells was calculated relative to NTCg1-targeted T cells. Error bars denote SEM. ** denotes p ≤ 0.01, as determined by an unpaired, two-tailed student’s T test. (TIF) [file ppat.1008305.s001.tif]

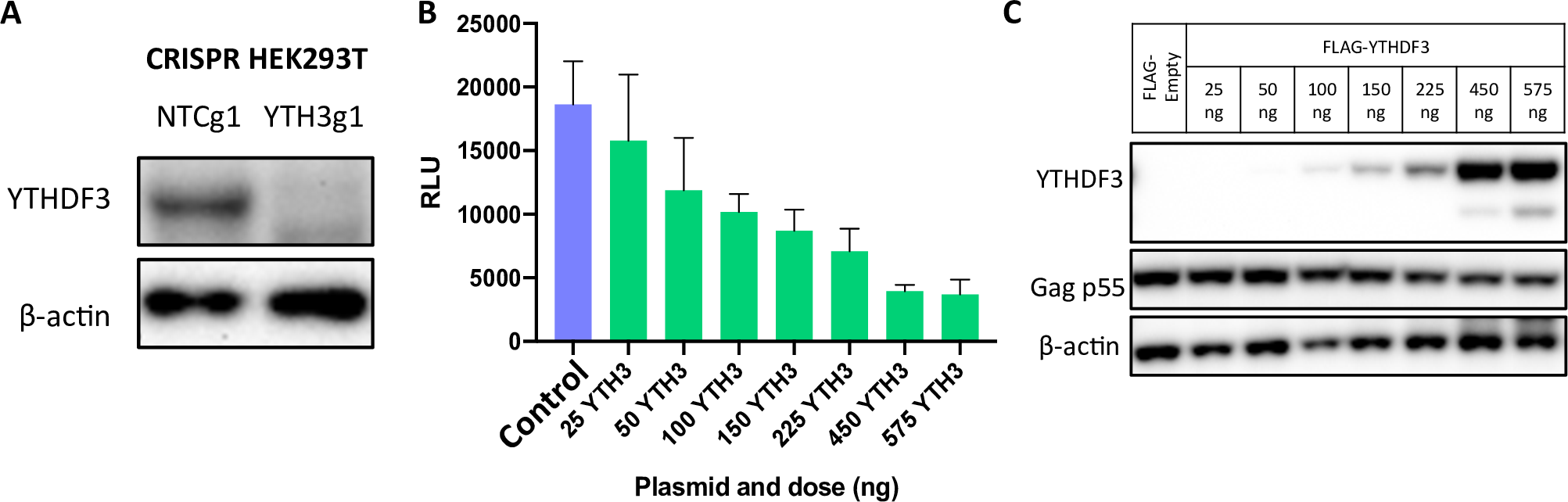

Supplement: S2 Fig — (A) Western blot of HEK293T-ΔYTHDF3 with anti-YTHDF3 ab103328 and anti-beta actin. YTHDF3 was knocked out using a CRISPR-Cas9 genome editing approach. (B) YTHDF3 expression negatively regulates HIV infectivity. Transfections were performed in HEK293T-ΔYTHDF3 cells with increasing amounts of FLAG-YTHDF3 plasmid (25–575 ng) in biological triplicates. TZM-bl reporter cells were infected with 5ul of viral supernatant. Data shown is representative of two independent experiments. (C) Western blot of the HEK293T-ΔYTHDF3 producer cells from which the viruses shown in S2C Fig were collected. Membranes were probed with anti-YTHDF3 ab103328, anti-p24 (Gag) and anti-beta actin. The Western blot is representative of two independent experiments. (TIF) [file ppat.1008305.s002.tif]

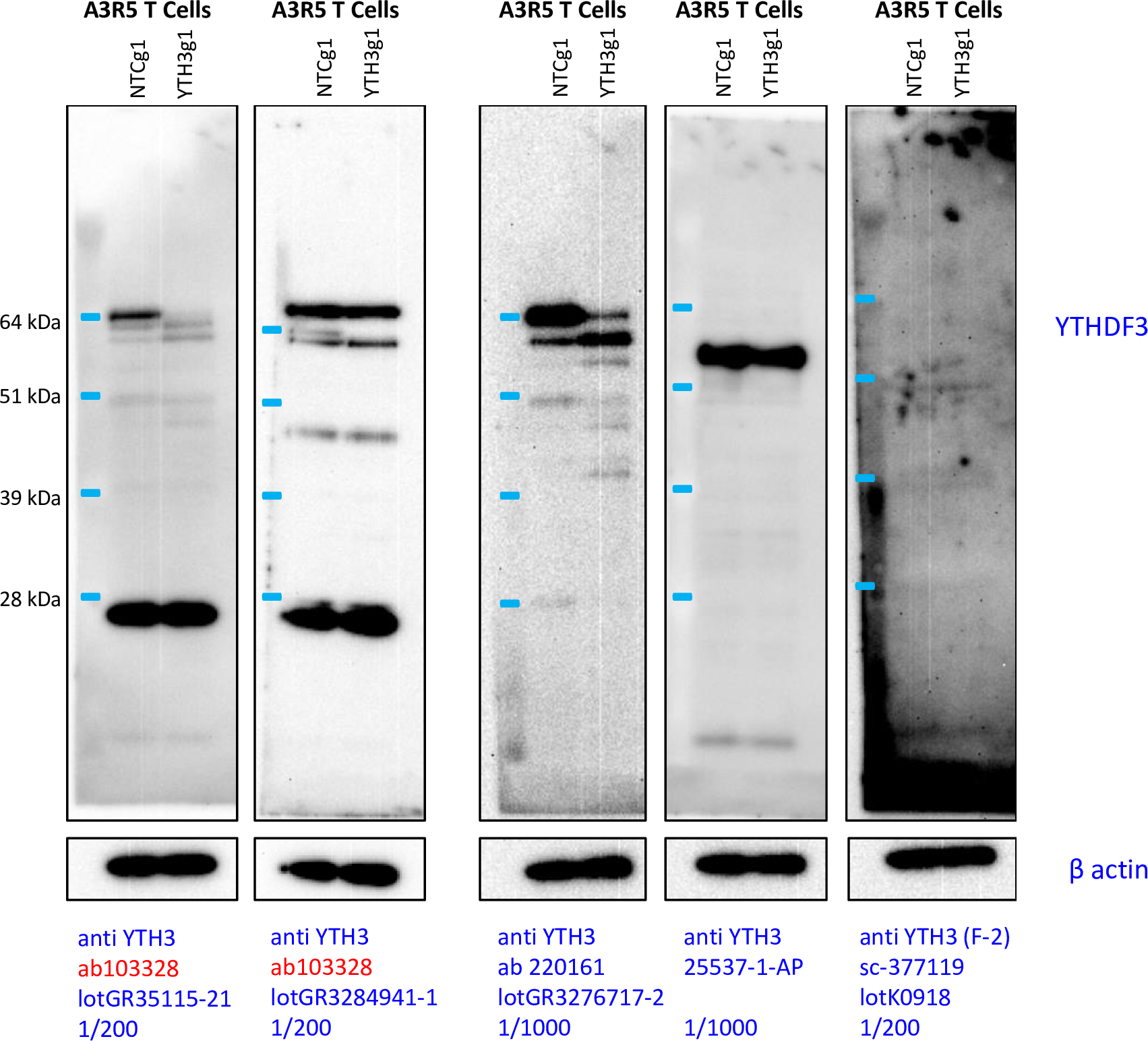

Supplement: S3 Fig — A panel of four different commercially available YTHDF3 antibodies and two different lots of the same YTHDF3 antibody were used to probe for endogenous YTHDF3 using cell lysates from A3R5-Rev-GFP NTCg1 and A3R5-Rev-GFP YTHDF3g1 T cells. Cellular YTHDF3 is detected at 64 kDa. Anti-beta actin was used as a loading control. (TIF) [file ppat.1008305.s003.tif]
